# 

**Published:** 1917-12-15

**Authors:** 


### CONTENTS.

| | PAGE |
|---|---|
| **Our Food and Ourselves** ... ... ... ... | 215 |
| **Some Penal Cases** ... ... ... ... ... | 216 |
| **Hospital and Institutional News** ... ... ... | 217 |
| Hospitals After the War—Does a Hospital Social Service Department Pay?—A Chairman on the Outlook—Baby Welfare—Swansea Orphan Home—The Fruits of Criticism—*Truth's* Christmas Number—British Troops in Durban—The Cost of a Nurse—The Combating of Venereal Diseases—Should a Hospital Tell?—Compulsory Rationing in Germany—Wigan's New Superintendent—Surgical Appliances for School Children—A Success in North Staffordshire—The Bradford Institute for the Blind—The Sale of Babies—A Christmas *Gazette*—This Week's Drug Market. | |
| **War in the Air** ... ... ... ... ... ... | 221 |
| The Medical Problems of Flying. | |
| **Palestine and its Hospitals** ... ... ... | 222 |
| Work at Gaza. | |
| **An Organiser and Administrator** ... ... | 223 |
| The Late Captain H. S. Tunnard. | |
| **Hospitals and the Christmas Tradition** ... | 224 |
| Their Place in the War Industries To-day. | |
| **Hospital Needs and Hospital Appeals** ... ... | 226 |
| **Tommy in Hospital** ... ... ... ... ... | 227 |
| The Third London Artists and their Work. | |
| **The Editor's Letter-Box** ... ... ... ... | 229 |
| The Churches and Venereal Marriages—A Medical Officer's Libel Action—Lady Roberts' Field Glass Fund. | |
| **Nursing Affairs in Ireland** ... ... ... ... | 230 |
| **The Matrons' and Sisters' Department** ... | 231 |
| Nursing Progress and its Developments. | |
| Round the Hospitals. | |
| The R.B.N.A. and the College—Why 16 Nurses Upset the Apple-cart—The Pity of it—How Working Nurses can Secure State Registration—A Neck-and-neck Race—Who will Win it? | |
| **The Fourth War Christmas** ... ... ... | 235 |
| Its Celebration in some Representative Institutions. | |
| **Parliament and the Profession** ... ... ... | 236 |
| **The Institutional Worker** ... .. (Suppl.) | 1 |
| An Economy Campaign in Hospitals. | |
| Question for December. | |
| Questions Invited. | |

